# Satisfaction of lecturers and undergraduate students of medical faculties in Indonesia towards online anatomy learning during COVID-19 pandemic

**DOI:** 10.1186/s12909-024-05620-x

**Published:** 2024-06-26

**Authors:** Isabella Kurnia Liem, Ayu Eka Fatril, Firda Asma’ul Husna

**Affiliations:** 1 Department of Anatomy, Faculty of Medicine, Universitas Indonesia, Jl. Salemba Raya 6, Jakarta, 10430 Indonesia; 2 Faculty of Medicine, Indonesia Museum of Health and Medicine, Indonesian Medical Education and Research Institute, Universitas Indonesia, Jl. Salemba Raya 6, Jakarta, 10430 Indonesia

**Keywords:** Online class, Anatomy learning, Undergraduate medical students, Anatomy lecturers, COVID-19

## Abstract

The changing of education activities (offline into online) to reduce coronavirus transmission during COVID-19 pandemi has influence on the learning strategies, which ultimately might impact the achievement of learning objectives. Therefore, we conducted a cross-sectional study using a valid (*p* < 0.01; two-tailed Pearson correlation) and reliable (*r* = 0.878 and *r* = 0.849; Cronbach Alpha) online questionnaire to evaluate the perspectives of human anatomy lecturers and undergraduate medical students in Indonesia in implementing the online anatomy learning. We also explored their expectations and preferred learning methods after COVID-19 pandemic. Using purposive sampling, 467 respondents, which consisted of 66 lecturers from 41 universities in six islands (Java, Sumatera, Kalimantan, Sulawesi, Nusa Tenggara and Papua) and 401 students from 19 universities in four islands (Java, Sumatera, Kalimantan, Papua) were recruited. A Chi-square test was used to analyze the differences in categorical variables. The lecturers (74.2%) and students (63.1%) agreed that online learning effectively delivered the course material (*p* = 0.095). They (69.7% lecturers and 57.9% students) also agreed that learning time allocation was sufficient (*p* = 0.079); moreover, lecturers (53%) and students (56.1%) had good interaction (*p* = 0.689). Nevertheless, 56.1% lecturers and 63.3% students had problem during online practical sessions. They had different perspectives about issues during online classes (69.7% vs 36.4%; *p* < 0.01), motivation improvement (72.7% vs 37.4%; *p* < 0.01), and time management (87.9% vs 58.4%; *p* < 0.01). According to the location of the university, especially in the student's side, students in Java had higher proportion in the two aspects, i.e. learning material (*p *< 0.01) and lecturer-student interaction (*p* < 0.01), and had lower proportion in the problems during online class (*p* = 0.003) and practical sessions (*p* = 0.008). Majority of the respondents (62,2% lecturers in Java, 71.4% lecturers outside Java, 79.6% Students in Java, and 76.6% students outside Java) preferred the use of blended learning after the pandemic (new normal era) and expected to continue the cadaveric practical sessions (82.2% lecturers in Java, 81.0% lecturers outside Java, 91.1% students in Java, and 78.3% students outside Java). In conclusion, the study showed that the satisfaction toward online anatomy learning related to the subject’s role (lecturer or student) and the university region in some aspects.

## Background

The COVID-19 pandemic has been occurring for more than two years and various variants have emerged, leading to the increased number of cases worldwide. Some variants are also able to penetrate self-protection after vaccination or natural infection [1]. The COVID-19 pandemic has easily spread to every continent, severely disrupting and affecting health, economy, psychology as well as education due to the strict lockdown [2]. In the education sector, during the peak of the pandemic, all schools, colleges, and universities were closed. Nevertheless, the learning goals have to be achieved; therefore, to cope with the situation, an online learning method was developed.

The online learning method was applied in all levels of education, subjects and courses, including in medical education. Undergraduate medical courses, including human anatomy were performed online, in a synchronous and/or asynchronous mode using web-based learning systems; video conference platforms such as Zoom Meeting, Google Meet, or Microsoft Teams; and social media platforms such as WhatsApp and Instagram. As a compulsory subject in medical curriculum, Anatomy course covering 12 subjects/blocks of human body systems must be completed by all undergraduate medical students [3]. In the national meeting of Indonesian Anatomist Association (IAA) on May 4–5, 2018 held in Jakarta, two learning objectives of anatomy learning have been formulated [4]. The first objective is that the students comprehensively understand human anatomy to acquire a strong basis for understanding the processes related to physiological and pathological changes. The second is that students can explain topics related to human anatomy to the general community using lay language [4]. Ideally, face-to-face learning is needed to achieve these two learning objectives. However, with the use of online learning systems during COVID-19 pandemic, the effectiveness of the recently developed systems to accomplish the objectives should be evaluated.

Online learning systems have been proven to be effective in suppressing the COVID-19 transmission rates between students; however, the impact on the achievement of education goals has been questioned, especially in medical education. A survey by Wijayanengtias and Claretta supported the fact that online learning was quite effective in reducing the transmission rate of COVID-19. Nevertheless, students were found dissatisfied with various aspects, such as supporting facilities and infrastructures, limitation in internet access, and the increased number of study assignments [5]. Those findings were consistent with the study results of Niemi and Kousa, which showed that the majority of students tended to feel more burdened, even suffered from headache and learning demotivation [6]. On the contrary, many lecturers claimed that online learning had a lot of positive effects, especially in the pandemic era. Nonetheless, lecturers did not deny that the online method was less comfortable. It also has limitations in the lecturer-student interaction and challenges in student assessments [7]. These contradictory statements from students and lecturers and the lack of studies about the implementation of online anatomy learning in Indonesia have led us to conduct an evaluation on the online anatomy learning during COVID-19 pandemic in Indonesia by identifying the lectures and students perspectives about the implementation of online learning methods in Indonesia, and their expectation for the learning methods in the new normal era (after the pandemic).

## Methods

### Research design

This research was a cross-sectional study designed to evaluate the satisfaction of Indonesian anatomy lecturers and students with the online learning through understanding the relationship between the role of the subjects (lecturer or student) and the university region (Java Island or outside Java Islands) with their perspective on online anatomy learning during COVID-19 pandemic and their expectations about the learning methods after the pandemic.

### Materials and/or subjects

#### Subjects

Anatomy lecturers (members of IAA) and undergraduate medical students from 88 medical faculties in Indonesia were invited to participate in this study. In 2020, there were 127,650 students of health higher education Health Students in Indonesia (Higher Education Statistics) [8], and 255 anatomy lecturers who were members of the communication forum on the Indonesian Anatomy WhatsApp group as the population for this study. To meet the need for research subjects who had experience in online learning and were active in anatomy teaching and learning, we set criteria for lecturers and students and obtained research subjects using non-probability sampling technique (purposive sampling). The inclusion criteria for the anatomy lecturers were currently teaching in medical faculties in Indonesia and had experience in online teaching; whereas, the inclusion criteria for the students were being registered at the undergraduate program of medical faculties in Indonesia and had experienced online learning for human anatomy courses.

A total of 467 respondents were recruited, including 66 lecturers from 42 universities in six islands (Java, Sumatera, Kalimantan, Sulawesi, West Nusa Tenggara and Papua) and 401 students from 19 universities in four islands (Java, Sumatera, Kalimantan, and Papua). Since the research focuses on nine aspects, based on sample to item(s) ratio which has a rule that the ratio should be at least 5:1 or more preferable 20:1 [9], the minimum sample size was in the range of 45–180.

This research has been approved by the Ethics Committee of the Faculty of Medicine Universitas Indonesia – Cipto Mangunkusumo Hospital, reference number: KET-979/UN2.F1/ETIK/PPM.00.02/2020. All the method were performed in accordance with the relevant guidelines and regulations. All respondents had agreed to be involved as research subjects by signing the informed consent; moreover, all respondents were 17 years old or older; therefore, they could sign their own informed consent, which was included in the Google Form questionnaire.

#### Materials

An online questionnaire was developed on the Google Form platform. The questionnaire was distributed via WhatsApp group of IAA from August until October 2020. The validity and reliability of the questionnaire were test in 30 respondents and analyzed using two-tailed Pearson correlation and Cronbach Alpha test, supported by the IBM Statistical Package for Science (SPSS) Statistics version 26 (IBM Corp.) software. Both questionnaires for lecturer and student were valid (*p* < 0.01) and reliable (*r* = 0.878 for lecture’s questionnaire and *r* = 0.849 for student’s questionnaire). Participation was voluntary and subject confidentiality was maintained. There were nine aspects being surveyed, which covered the satisfaction toward online anatomy learning during COVID-19 pandemic (seven aspects with agree/disagree response options) and the expectation of anatomy learning methods and activities after the pandemic or new normal era (two aspects with closed questions).

#### Technical information

This research was carried out by adapting a research method initiated by Pather et al*.,* involving some procedures, i.e., data overview, preliminary coding, theme defining, theme review, theme naming, theme summary and report, questionnaire validation, and questionnaire distribution [10].

#### Statistical analysis

The characteristics summary of the respondents are displayed as percentage values in one table. A Chi-square test was used to analyze the differences in categorical variables, i.e. the subject’s role (lecturers or students) and location of the university (Java or outside Java Island) related to their perspectives (agree or disagree) toward seven aspects of satisfaction towards online anatomy learning. The difference was considered significant if the *p*-value was < 0.05. The other two aspects, that is the expectations of the lecturers and students on anatomy learning methods and the expectations of the lecturers and students about learning activities needed to be repeated in the new normal era based on Indonesian regions were display descriptively as percentage diagram. Microsoft Excel (Version 16.78.3 [23102801]; Retail License 2019; ©2023 Microsoft) and IBM SPSS Statistics version 26 (IBM Corp.) were used to analyze the data.

## Results

A total of 467 respondents joined the survey, which consisted of 66 human anatomy lecturers and 401 undergraduate students of medical faculty in Indonesia (Table 1). The lecturers recruited were affiliated to 41 universities (46.6% of all medical faculties in Indonesia) and resided in Java, Sumatera, Kalimantan, Sulawesi, Nusa Tenggara and Papua. The lecturers in this study represented a wide range of age, i.e., between 27–71 years with an average of 41.0 (SD 9.4) years. The ratio between male and female lecturers was 1:1.4. They had on average 11.8 (SD 9.0) years of experience as an anatomy lecturer. The students were the second, third and fourth grade medical students from 19 universities in Java, Sumatera, Kalimantan, and Papua. The students’ ages ranged from 17 to 28 years, with an average of 19.7 (SD 1.3) years. The ratio between male and female students was 1:2.3.

**Table 1:** Characteristics of research subjects

Characteristics	Categories	Java [n (%)]	Outside Java [n (%)]	Total [n (%)]
**Lecturers (*N* = 66)**				
Gender	Female	28 (62.2%)	10 (47.6%)	38 (57.6%)
	Male	17 (37.8%)	11 (52.3%)	28 (42.4%)
Age	≤ 45 years	27 (60.0%)	17 (81.0%)	44 (66.7%)
	> 45 years	18 (40.0%)	4 (19.0%)	22 (33.3%)
Teaching duration	≤ 10 years	18 (40.0%)	14 (66.7%)	32 (48.5%)
	> 10 years	27 (60.0%)	7 (33.3%)	34 (51.5%)
**Students (*N* = 401)**				
Gender	Female	111 (70.7%)	168 (68.8%)	279 (69.6%)
	Male	46 (29.3%)	76 (31.2%)	122 (30.4%)
Age	≤ 20 years	138 (87.9%)	190 (77.9%)	328 (81.8%)
	> 20 years	19 (12.1%)	54 (22.1%)	73 (18.2%)
Class	2	94 (59.9%)	85 (34.8%)	179 (44.6%)
	3	55 (35.0%)	106 (43.5%)	161 (40.2%)
	4	8 (5.1%)	53 (21.7%)	61 (15.2%)

*N* Total subject, *n* Number of subjects within a group

### Lecturers and students satisfaction towards online anatomy learning during COVID-19 pandemic

Most of the lecturers perpective about online anatomy learning during COVID-19 pandemic showed no relation with their university location. Six out of seven aspects about online anatomy learning during COVID-19 pandemic showed no proportion differences (*p* > 0.05) between lecturers in Java (*N* = 45) and outside Java (*N* = 21) (Table 2). Most of the lecturers agreed that they could manage their time well, had sufficient learning time allocation, and learning material was well delivered and understandable. They also did not find any issues during online classes as well as online practical sessions. In addition, they agreed that online classes improved students' motivation. However, they had different perspectives about the lecturer-student interaction during online learning (*p* = 0.029). More than 62% of the lecturers in Java agreed that lecturer-student interaction was good during online learning. On the other hand, more than 66% of lecturers outside Java did not agree with this (Table 2).

**Table 2:** Proportion of lecturers and students satisfaction towards online anatomy learning during COVID-19 pandemic based on regions in Indonesia

No	Aspects	Perspective	Lecturers (*N* = 66)			Students (*N* = 401)		
			**Java (*N* = 45)** **[n (%)]**	**Outside Java (*N* = 21)** **[n (%)]**	***p*-value**	**Java (*N* = 157)** **[n (%)]**	**Outside Java (*N* = 244)** **[n (%)]**	***p*-value**
1	Materials were well delivered and understandable	A	36 (80.0)	13 (61.9)	0.117	116 (73.9)	137 (56.1)	***p* < 0.01**
		DA	9 (20.0)	8 (38.1)		41 (26.1)	107 (43.9)	
2	No issues were found during online classes	A	31 (68.9)	15 (71.4)	0.834	71 (45.2)	75 (30.7)	**0.003**
		DA	14 (31.1)	6 (28.6)		86 (54.8)	169 (69.3)	
3	No issues were found during online practical sessions	A	23 (51.1)	6 (28.6)	0.086	70 (44.6)	77 (31.6)	**0.008**
		DA	22 (48.9)	15 (71.4)		87 (55.4)	167 (68.4)	
4	Online class improves student motivation	A	36 (80.0)	12 (57.1)	0.052	64 (40.8)	86 (35.2)	0.265
		DA	9 (20.0)	9 (42.9)		93 (59.2)	158 (64.8)	
5	Good interaction between lecturers and students	A	28 (62.2)	7 (33.3)	**0.029**	113 (72.0)	112 (45.9)	***p* < 0.01**
		DA	17 (37.8)	14 (66.7)		44 (28.0)	132 (54.1)	
6	Sufficient learning time allocation	A	32 (71.1)	14 (66.7)	0.714	100 (63.7)	132 (54.1)	0.580
		DA	13 (28.9)	7 (33.3)		57 (36.3)	112 (45.9)	
7	Respondents could manage their time well	A	41 (91.1)	17 (81.0)	0.239	98 (62.4)	136 (55.7)	0.185
		DA	4 (8.9)	4 (19.0)		59 (37.6)	108 (44.3)	

*Chi-square test* significant proportion difference between groups is shown by the *p*-value < 0.05, *A* Agree, *DA* Disagree, *N* Total subjects, *n* Agree or disagree subjects within a group

From the student side, it appears that the perspective about online anatomy learning are related to the location of the university. Students in Java (*N* = 157) have higher proportion of agreement that the material were well delivered and understanable (73.9%; *p* < 0.01), as well as their perception about interaction between lecturers and students (72.0%; *p* < 0.01). Student outside Java Island (*N* = 244) agreed that there were issues found in the online learning classes (69.3%; *p* = 0.003) and practical sessions (68.4%; *p* = 0.008). Nevertheless, most of students from Java and outside Java agreed that they had sufficient learning time allocation and could manage their time well, and did not agree that online learning improved their motivation (Table 2).

Three aspects of online anatomy learning, i.e. issues during online classes, improvement of student’s motivation, and time management are significantly related to the role of the subjects (Table 3). Most lecturers agreed that there were no issues found during online classes (69.7%) and online learning could improve student motivation (72.7%); however, it was in contrast to the students’ perspective (*p* < 0.01), with only 36.4% and 36.7% students agreed to the earlier statements, respectively. Nevertheless, the majority of lecturers and students agreed that they could manage their time well, even though there was a difference in the agreement proportion (*p* < 0.01) 87.9% and 58.4% respectively). On the other aspects, more than 50% of them agreed that during online learning they could have a good interaction and that the learning time allocation was sufficient (Table 3).

**Table 3:** Proportion comparison of Indonesian lecturers and students’ satisfaction towards online anatomy learning during COVID-19 pandemic

No	Aspects	Perspective	Lecturers (*N* = 66) [n (%)]	Students (*N* = 401) [n (%)]	*p*-value
1	Materials were well delivered and understandable	A	49 (74.2)	253 (63.1)	0.095
		DA	17 (25.8)	148 (36.9)	
2	No issues were found during online classes	A	46 (69.7)	146 (36.4)	***p* < 0.01**
		DA	20 (30.3)	255 (63.6)	
3	No issues were found during online practical sessions	A	29 (43.9)	147 (36.7)	0.275
		DA	37 (56.1)	254 (63.3)	
4	Online class improves student motivation	A	48 (72.7)	150 (37.4)	***p* < 0.01**
		DA	18 (27.3)	251 (62.6)	
5	Good interaction between lecturers and students	A	35 (53.0)	225 (56.1)	0.689
		DA	31 (47.0)	176 (43.9)	
6	Sufficient learning time allocation	A	46 (69.7)	232 (57.9)	0.079
		DA	20 (30.3)	169 (42.1)	
7	Respondent could manage their time well	A	58 (87.9)	234 (58.4)	***p* < 0.01**
		DA	8 (12.1)	167 (41.6)	

*Chi-square test* significant proportion difference between groups is shown by the *p*-value < 0.05, *A* Agree, *DA* Disagree, *N* Total subjects, *n* Agree or disagree subjects within a group

### Lecturers and students expectations about learning activities in the new normal era

**Fig. 1:**
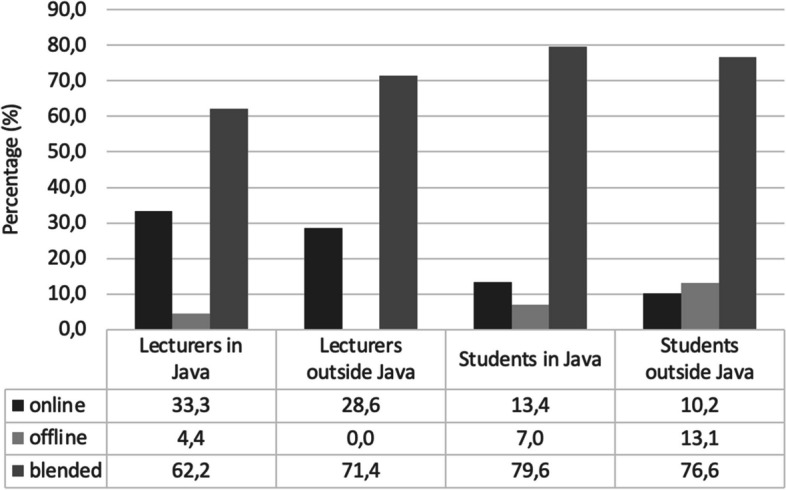
Expectations of the lecturers and students on anatomy learning methods in the new normal era based on Indonesian regions

**Fig. 2:**
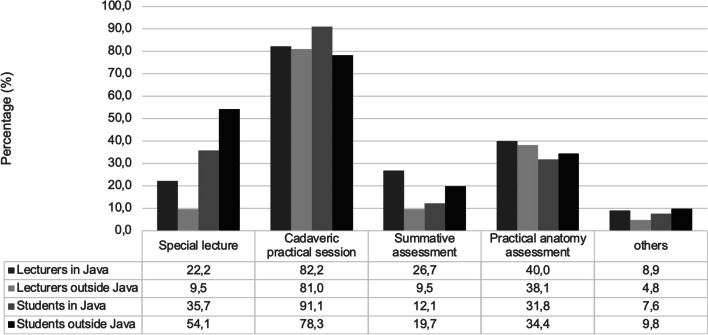
Expectations of the lecturers and students about learning activities needed to be repeated in the new normal era based on Indonesian regions

Being asked for their expectations of the anatomy learning methods after the pandemic subsides (the new normal era) (Fig. 1), a majority of lecturers and students in Java and outside Java expected to have a combination of online and offline classes (blended learning). For the learning activities, the majority of lecturers and students from Java and outside Java had the same expectation that they need a repetition of the cadaveric practical sessions (Fig. 2), without repeating the assessments. Among students from outside of Java, the majority (54.1%) felt that they needed a repetition of selected topics (special lectures) on anatomy.

## Discussion

Our study on the satisfaction of Indonesian anatomy lecturers and medical students on online anatomy learning showed that generally, most of them were satisfied in three aspects, i.e., learning materials were well delivered and understandable, sufficient time allocation, and good time management. This perceptions were not related to the role of subject and their university location. Differ with the offline method, online learning enables the lecturers and students to face the monitor and share contents via share screen. Therefore, the learning materials can be accessed better. Moreover, the learning interaction can be recorded, which allows students or lecturers to re-watch it if needed. These results is in agreement with Light and Pierson*,* who affirmed that online classes could facilitate learning material distribution effectively [11]. Satisfaction in time allocation and time management were thought to be associated with the prime characteristic of online class, namely, flexibility [12]. Moreover, online classes allow students to be able to understand materials at any time they prefer and to discuss them later with peers [13].

There were proportion differences in the perspectives of lecturers and students regarding whether there were problems in implementing online classes (lectures). The perception that there was no issues occurred during online classes had higher proportion in the lecturers (69.7%; *p* < 0.01). Consistent results were seen in the data based on Indonesian regions. Challenges in performing online learning are the internet connection quality and poor utilization skills in online technology support [14]. There were some typical issues usually faced by students when attending online classes, such as less intensive communication, overloaded tasks, unskillful use of technological tools, anxiety and stress (probably due to the pandemic situation), physical problems, and technophobia [15, 16]. This difference in perspectives arose because lecturers seemed to have a difficulty in assessing students’ learning progress during online classes [6]. Incapacity for that process had disallowed lecturers to identify any issues that hindered student progress during online classes. Thus, it is recommended to provide tutor and student guidelines for online teaching and learning activities, including any aspects related to the student learning process during online learning.

Perception that anatomy practical sessions were still problematic did not relate to the subject’s role, but related to the region in Indonesia, especially in the students side. A plausible reason for this phenomenon is the hands-on nature of the anatomical course [17]. Conducting online practical sessions is a challenge for lecturers, because they must prepare audiovisual learning media while previously, they should only use mannequins or cadavers. More than half of the students (63.3%) admitted that a number of issues arose during online practical sessions, due to the students’ inability to experience real-life anatomy and to perceive how the actual organs are located in the human body. Study of Potu et al. showed that the majority of the students (57.4%) preferred offline demonstration to understand the spatial orientation of the organ systems and to get a visualization of relations between structures [12]. In fact, cadaver is one of the most important practical media to help students learn about the human body system in reality, as it provides 3D visualization. Using cadavers is highly expected to effectively help prospective doctors enhance their surgery skill, professionalism, recognition of real human body organs, and teamwork [18]. The pandemic caused an absence of cadaveric dissection practical sessions. According to Ooi and Ooi, anatomy practical sessions without the use of cadavers might cost students lost opportunities for doctor’s professional skill development, professionalism enhancement, ethical competence improvement, and self-confidence acquisition when working in surgery rooms. Besides, the absence of cadaveric media for practical sessions might also cause a loss of chance for students to sharpen their teamwork skill. Because of these potential effects, students and lecturers admitted that they were less satisfied with the online practical exercises, which lead to a consideration in planning the prospective practical sessions in the new normal era, by reincluding the use of cadaveric media.

Next aspect is related to the improvement of student’s interest in online classes. Lecturers had higher proportion (72.7%) in opined student’s interest increased during online class. This was inverted with the students perceived. A total of 62.6% of students disagreed that online classes had motivated them to learn. These results were consistent with the results based on regions. These findings are also consistent with the research carried out by Adnan and Anwar, who found that 74% of students disagreed that online classes were more motivating than conventional ones [19]. This perception appears to be the reason online classes might demotivate students to learn. Yunitasari and Hanifah found that the COVID-19 pandemic affected student’s motivation as they felt bored and could not be involved in live interaction that was experienced in face-to-face learning [20]. Another probability causing a student's demotivation is overloaded projects/tasks. Survey on 68 medical students in Germany had demonstrated how students became so permanently stressed during COVID-19 due to excessive emotional fatigue, especially when examination was approaching. It forced them to spend the whole time studying, and sacrifice other routines, including their time for rest [21]. Moreover, online class is also a cause of excessive anxiety and a source of stress to students. Alsoufi et al*.* asserted that around 31.3% of medical students in Libya were highly prone to depression, and the other 10.5% suffered from anxiety symptoms [22]. More seriously, it was also reported that most students were demotivated to learn during the pandemic [6].

Interesting results were found in the aspect of interaction between lecturers and students. This was not related to the subject’s role, but related to the region in Indonesia. In general, more than 50% respondents agreed that during the online anatomy learning, lecturers and students had a good interaction. However, based on the regions, respondent from Java had higher proportion compare to the outside Java. The most probable cause was the level of internet penetration in Indonesia. In 2020–2021, Java was the island with the highest number of villages that were able to receive cellular phone signals and with a strong signal reception in Indonesia, amounting to 99.96%, followed by Sumatera (98.61%), Bali and Nusa Tenggara (98.63%), Sulawesi (95.79%), and Kalimantan (93.60%). Maluku and Papua were the regions with the fewest signal receiving villages [23]. In online activities, internet signal strength and the availability of receiving devices (such as smartphones and computers) are prerequisites for the smooth running of learning activities. Further, most of the lecturers also felt uncomfortable with online classes, which was the cause of ineffective interaction, as well [7]. Similar conditions have also been reported in India. Kumari et al. reported that a majority of students in the Medical College of Jharkhand India experienced network issues (53.89%), whereas, Sarkar et al. with respondents from various universities in India reported internet difficulties experienced by 42.06% of the students. Other hindrance factors, such as personal communication skill, direct communication limitation, and other reasons can also be potential factors that are needed to be evaluated [16, 24, 25].

After the pandemic, both lecturers and students chose a combination of online and face-to-face learning (blended learning) to be carried out and continue the practical learning activities using cadavers. Respondents from Java thought that they needed a repetition of learning anatomy, especially the practical anatomy sessions. Whereas outside Java, apart from repeating the practicum, they also need additional lectures on certain topics (special lectures). The decision might be directed by the fact that both online and offline (face-to-face) learning activities have their advantages and disadvantages. From this research, the majority of respondents agreed that online learning had advantages, i.e. materials were well delivered and understandable, time could be allocated sufficiently and manageable (more flexible). Nevertheless, there were disadvantages, such as limitation of lecture and student interactions, problems in its implementation, both for online classes and practical sessions. These issues will finally influence the motivation of the students. Low student motivation and less optimal interaction among students become the key reasons that encouraged students and lecturers to choose mixed methods, combining online and offline modes. Similarly, previous research reported by Potu et al. that 57.40% and by Rajab et al. that 62.5% of students agreed to undertake a blended learning model [12, 15]. Furthermore, there were results of surveys in which the respondents chose online or face-to-face learning only. Thomas et al*.* claimed that about 55% of students preferred conventional classes to the online ones, and 51.7% of students were found not expecting to rejoin online learning after the pandemic is over [26]. In Lahore, Fatima et al. reported that 59.5% of respondents valued face-to-face learning more than online learning. In India, it was reported that 68.86% [24] to 98.41% [16] of respondents agreed that traditional teaching was better than online anatomy teaching.

The COVID-19 pandemic has caused changes in the majority of life sectors, especially education. Before the pandemic, education was normally held in a traditional setting – face-to-face. Such method allowed students and lecturers to interact with one another to develop favorable social interaction between the two. Furthermore, social interaction could generate active participation in team working, allow students to help each other in doing the assignment, and understand each other through facial gestures, so that essential points of learning could be acquired [27]. Unfortunately, the pandemic changed everything, and such a normal activity could not be held anymore. Therefore, in response to the current condition, modification on the learning method used is highly needed, by combining online and offline class activities. Basically, the implementation of exercises in online classes needs to consider some crucial aspects, such as technology, finance, infrastructure, institution, educators, internet connection quality, and family distraction, which possibly gives impact to the success of online classes [13, 28]. Further, a number of research demonstrated positive results on online classes. In fact, the majority of students preferred online to offline classes due to its flexibility, so that learning could be accomplished anytime and anywhere [29]. It was also supported by Febrianto et al., who claimed that efficiency and flexibility were the major reasons why online class was promoted [30]. In addition, with online classes, students could still have open access to skill and knowledge enhancement without having to worry about the risk of coronavirus infection [31].

## Conclusions

This study showed that satisfaction towards online anatomy learning during COVID-19 related to the subject’s role in three aspects, i.e. time management, student motivation, and problems during online classes. It did not related to the four aspects, i.e. learning material, problems during online practical sessions, lecturer-student interaction, and time allocation. Satisfaction towards online anatomy learning were much related to the university region, especially on the students side. Most of students in Java had higher satisfaction compare to the students outside Java; namely, higher proportions in the two aspects, i.e. learning material and lecturer-student interaction, and had lower proportion in the problems during online class and practical sessions. A majority of the respondents preferred the use of blended learning after the pandemic (new normal era) and expected to continue the cadaveric practical sessions. This research not only shows us that online learning really has a positive effect on the teaching and learning process, but also indicates that face-to-face learning is still needed, especially as the use of cadavers cannot be eliminated in anatomy learning. Therefore, in line with the expectations of respondents in this research, after the pandemic, blended learning is the best method to try to implement.

## Data Availability

The datasets used and/or analyzed during the current study are available from the corresponding author on reasonable request.
